# Supplementary material for: Selective EMC subunits act as molecular tethers of intracellular organelles exploited during viral entry
Source: Nat Commun. 2020 Feb 28;11:1127. doi: 10.1038/s41467-020-14967-w (PMC7048770; doi:10.1038/s41467-020-14967-w)
Supplement: Supplementary file 1 — Supplementary Information [file 41467_2020_14967_MOESM1_ESM.pdf]

Selective EMC subunits act as molecular tethers of intracellular organelles exploited during viral entry

Bagchi et al.

Supplementary Information

Supplementary Methods:

Transmission Electron Microscopy (TEM)

CV-1 cells were infected with SV40, fixed chemically (2.5% glutaraldehyde, 4% paraformaldehyde, 0.02% picric acid, 1% osmium tetroxide) at 5 hours post infection, dehydrated with ethanol, and embedded in Durcupan ACM Epoxy resin (Electron Microscopy Sciences, PA). The thin sections (70 nm in thickness) were cut using an ultramicrotome with a diamond knife. The sections were collected onto either bare copper grids and contrast stained with salts of uranium (2% uranyl acetate) and lead (lead citrate 3%) (Electron Microscopy Sciences, PA) to reveal the ultrastructure of the cells and are finally viewed by TEM (JEOL JEM 1400 Plus).

Immuno-EM

EMC4-deleted COS-7 cells expressing EMC4-FLAG, or EMC7-depleted COS-7 cells expressing EMC7-FLAG, were infected with SV40, fixed chemically (3% paraformaldehyde, 0.2% glutaraldehyde) at 5 hours post infection, dehydrated with ethanol, and embedded in London Resin (LR) white (Electron Microscopy Sciences, PA) at 55°C for 48 hours. The thin sections (70 nm in thickness) were cut using an ultramicrotome with a diamond knife. The sections were collected onto bare nickel grids. The sections are then stained immunochemically with primary antibodies raised against antigens exposed on the surface of the sections. The primary antibodies are visualized by staining immunochemically with secondary antibodies raised against the species and isotype of the primary antibodies, conjugated to colloidal gold particles. The

immunochemically stained sections are then contrast stained with 0.05% osmium tetroxide, followed by salts of uranium (2% uranyl acetate) and lead (lead citrate 3%) (Electron Microscopy Sciences, PA) to reveal the ultrastructure of the cells, and are finally viewed by TEM (JEOL JEM 1400 Plus).

**Oligonucleotide Name**	**Sequence**
EMC4-FLAG F	5’- attgttgctagcatgacggcccaggggggcctggtggctaaccg- 3’
EMC4-FLAG R	5’- attgttggtaccttacttgtcgtcatcgtctttgtagtccaaaagcagtcctccaccactgaactccattctctca -3’
EMC6-FLAG F	5’- attgttgctagcatggccgcggtggtggccaagcgggaag -3’
EMC6-FLAG R	5’- attgttggtaccttacttgtcgtcatcgtctttgtagtcgtagacgtgcaccatgccgtagaggaacgtccagaa -3’
EMC7-FLAG F	5’- attgttctcgagatggcggccgctctgtggggcttctttcccgtcctgc -3’
EMC7-FLAG R	5’- attgttggatccctacttgtcgtcatcgtctttgtagtccctccttttgccagccccacttttgcctgttttactgctgccgctgcta -3’
EMC6 sires F	5’- gagaatgaggagcagggagagcaggacggaggcgagcaggtag -3’
EMC6 sires R	5’- aaggcgggaaggaggtggaataagtacttcaaatcacggagacctc -3’
EMC7 sires F	5’- cgggatcaaatctgtaagctggagatacaacttccactacataagatccagaaggtatatcatga -3’
EMC7 sires R	5’- ttcgagtggatatcacttcgaaaggaaagatgagggctagatatgtgaattacatcaaaac -3’
EMC7 del (218-239) F	5’- aaaaggagggactacaaagacgatgacgacaagtaa -3’
EMC7 del (218-239) R	5’- gagtcttgtcatgaactcagaaacatcaggcaactcatggttgg -3’
EMC7 del (188-201) F	5’- aattccaaccatgagttgcctgatgtttctgagttcatgacaagactc -3’
EMC7 del (188-201) R	5’- atcacttgtgttgaccactttaggcagaagcacaaatatcaata -3’
myc-BioID2-EMC4 F	5’- attgttctcgagacggcccaggggggcctggtggctaaccg -3’
myc-BioID2-EMC4 R	5’- attgttggtaccttacaaaagcagtcctccaccactgaactccattctctca -3’
EMC7-BioID2-HA F	5’- attgttgctagcatggcggccgctctgtggggcttctttcccgtcctgc -3’
EMC7-BioID2-HA R	5’- attgttaccggtacctccttttgccagccccacttttgcctgttttac -3’
GFP11-STARD3-FLAG F	5’-attgttctcgagatgagagaccacatggtgctgcacgagtacgtgaacgccgccggcatcaccatgagcaagctgcccagggagctgacccga -3’
GFP11-STARD3-FLAG R	5’- attgttggatccctacttgtcgtcatcgtctttgtagtccgcccgggcccccagctcgctgatgcgctgtcgcaggtgaaaggc -3’
GFP(1-10)pcDNA3.1(-) F	5’- gatccgctagccgccaccatggtttcgaaaggcgaggagctgttcacag -3’
GFP(1-10)pcDNA3.1(-) R	5’- attcctcgagttttctcgtttgggtctttgctcagcactgtctgtgtgctca -3’
GFP(1-10)-B14 F	5’- aaactcgaggaatggaggggaacagggatgaggctgag -3’
GFP(1-10)-B14 R	5’- atcggatcctcatcctcctttataaagactggtaagccgctctaa -3’

Supplementary Table 1: List of oligonucleotides

Supplementary Table 2: Key Resources Table

REAGENT or RESOURCE	SOURCE	IDENTIFIER
Antibodies		
Anti-BAP31	Thermo Fisher Scientific	Cat# MA1-34492; RRID: AB_2537133
Anti-EMC1	Abgent	Cat# AP10226b; RRID: AB_10817224
Anti-EMC4 (TMEM85)	Abcam	Cat# ab184162; RRID: AB_2801471
Anti-EMC6 (TMEM93)	Aviva Systems Biology	Cat# ARP44679_P050; RRID: AB_2048477
Anti-EMC7	Thermo Fisher Scientific	Cat# PA5-52688; RRID: AB_2641011
Anti-FLAG clone M2	Sigma Aldrich	Cat# F1804; RRID: AB_262044
Anti-FLAG	Sigma Aldrich	Cat# F7425; RRID: AB_439687
Anti-GFP	Proteintech	Cat# 660002-1-Ig; RRID: N/A
Anti-HA	Proteintech	Cat# 51064-2-AP; RRID: AB_11042321
Anti-SV40 large T antigen	Santa Cruz Biotechnology	Cat# sc-147; RRID: AB_628305
Anti-Hsp90	Santa Cruz Biotechnology	Cat# sc-13119; RRID: AB_675659
Anti-Stx18	Santa Cruz Biotechnology	Cat# sc-293067; RRID: AB_10647235
Anti-SV40 VP1 antibody (mouse monoclonal)	Gift from Walter Scott (University of Miami)	N/A
Anti-SV40 VP1 antibody (rabbit polyclonal)	Gift from Harumi Kasamatsu (UCLA)	N/A
Anti-BiP	Abcam	Cat# ab32618; RRID: AB_732737
Anti-SV40 VP2/3	Abcam	Cat# ab53983; RRID: AB_946339
Anti-CTA	EMD Millipore	Cat# 227040; RRID: AB_211712
Anti-Rab7 (mouse monoclonal)	Sigma Aldrich	Cat# R8779; RRID: AB_609910
Anti-Rab7 (rabbit monoclonal)	Cell Signaling Technology	Cat# 9367; RRID: AB_1904103
Anti-Protrudin (ZFYVE27)	Proteintech	Cat# 12680-1-AP; RRID: AB_10640298
Anti-EEA1 (rabbit monoclonal)	Cell Signaling Technology	Cat# 3288; RRID: AB_2096811
Anti-Myc	Gift from Kristen Verhey (University of Michigan), Santa Cruz Biotechnology	Cat# sc-40; RRID: AB_627268
Anti-Rab5	Abcam	Cat# ab18211; RRID: AB_470264

Anti-STARD3 (MLN64)	Abcam	Cat# ab3478; RRID: AB_303838
Anti-DnaJB14	Proteintech	Cat# 16501-1-AP; RRID: AB_2094414
F(ab') 2 Fragment of Goat-anti-Mouse IgG (H&L) (EM grade 15nm)	Electron Microscopy Sciences	Cat # 25377; RRID: N/A
F(ab') 2 Fragment of Goat-anti-Rabbit IgG (H&L) (EM grade 6nm)	Electron Microscopy Sciences	Cat # 25364; RRID: N/A
Goat-anti-Mouse IgG (H&L) (EM grade 6nm)	Electron Microscopy Sciences	Cat # 25124; RRID: N/A
Goat-anti-Rabbit IgG (H&L) (EM grade 15nm)	Electron Microscopy Sciences	Cat # 25113; RRID: N/A
Bacterial and Virus Strains		
SV40	Lab Stored	Inoue and Tsai, 2011
Chemicals, Peptides, and Recombinant Proteins		
Deoxy Big Chap detergent	Millipore Sigma	Cat# 256455
DMEM	Thermo Fisher Scientific	Cat# 11995
HyClone FetalClone III Serum	Thermo Fisher Scientific	Cat# SH3010902
Trypsin-EDTA (0.25%)	Thermo Fisher Scientific	Cat# 25300054
Fugene HD	Promega	Cat# E2311
Lipofectamine RNAiMax	Thermo Fisher Scientific	Cat# 13778150
Penicillin-Streptomycin	GIBCO	Cat# 15140-122
Prolong Gold with DAPI	Thermo Fisher Scientific	Cat# P36941
Vectashield Antifade Mounting Medium	Vector Laboratories	Cat# H-1000
Triton X-100	Sigma Aldrich	Cat# 93443
Anti-FLAG M2 Affinity Gel	Sigma Aldrich	Cat# A2220
Dynabeads MyOne Streptavidin C1	Thermo Fisher Scientific	Cat# 65001
Streptavidin- HRP	Thermo Fisher Scientific	Cat# 434323
IGEPAL CA-630 (NP40)	Sigma Aldrich	Cat# I8896
Biotin	Sigma Aldrich	Cat# B4501
AURION BSA-C (acetylated BSA)	Electron Microscopy Sciences	Cat# 25557
Osmium Tetroxide (2% Aqueous Solution)	Electron Microscopy Sciences	Cat# 19152
Picric Acid Saturated, Aqueous	Electron Microscopy Sciences	Cat# 19552
16% Paraformaldehyde (formaldehyde) aqueous solution	Electron Microscopy Sciences	Cat# 15710
Glutaraldehyde (10% Aqueous Solution)	Electron Microscopy Sciences	Cat# 16120
2% Uranyl Acetate Solution	Electron Microscopy Sciences	Cat # 22400-2
Lead Citrate 3%, Ready to use	Electron Microscopy Sciences	Cat # 22410

Durcupan ACM Epoxy (Single Component A)	Electron Microscopy Sciences	Cat # 14041
Durcupan ACM Epoxy (Single Component B)	Electron Microscopy Sciences	Cat # 14042
London Resin White	Electron Microscopy Sciences	Cat# 14380
Sorensen's Phosphate Buffer	Electron Microscopy Sciences	Cat# 11600-05
Experimental Models: Cell Lines		
CV-1	ATCC	Cat# CCL-70
HEK 293T	ATCC	Cat# CRL-11268
COS-7	ATCC	Cat# CRL-1651
Oligonucleotides		
See Supplementary Table 1	N/A	N/A
Recombinant DNA		
pcDNA 3.1 (-)	Thermo Fisher Scientific	Cat# V79520
MCS-BioID2-HA	Addgene	Cat# 74224
Myc-BioID2-MCS	Addgene	Cat# 74223
EGFP-WT Rab7	Gift from Joel Swanson (University of Michigan)	N/A
EGFP-N125I Rab7	Gift from Joel Swanson (University of Michigan)	N/A
EGFP-Q67L Rab7	Gift from Joel Swanson (University of Michigan)	N/A
FLAG-EMC4	Gift from Andrew Tai (University of Michigan)	N/A
FLAG-Δ50 EMC4	Gift from Andrew Tai (University of Michigan)	N/A
Software and Algorithms		
FIJI/ImageJ	NIH	N/A
NIS-Elements AR	Nikon	N/A

Supplementary Figure 1. SV40 does not accumulate in the early endosome in EMC4- or EMC7-depleted cells. (related to Figure 3)

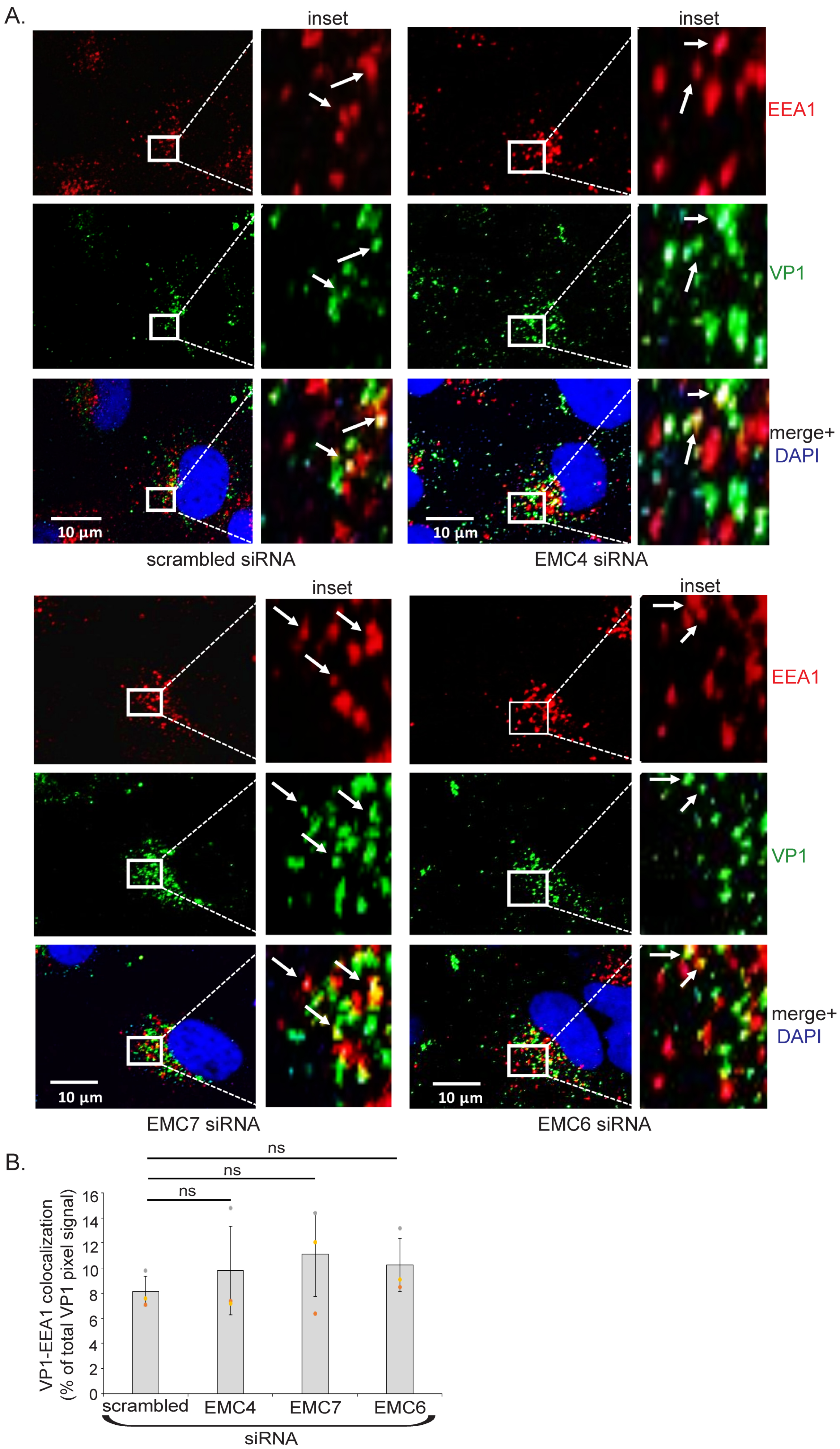

Supplementary Figure 1. SV40 does not accumulate in early endosome in EMC4- or EMC7 depleted cells. (related to Figure 3)

A. CV-1 cells transfected with the indicated siRNA were infected with SV40 (MOI ~10), fixed at 6 hours post infection, and stained with specific antibodies followed by confocal microscopy. DAPI positions the nucleus. B. Total pixels of VP1 and EEA1, as well as pixels positive for both VP1 and EEA1, were quantified by FIJI/ImageJ. The percentage of VP1-EEA1 overlapping (co-localized) pixels among total VP1 pixel signal in A is shown. The image analysis was done on a single z-plane. Values represent the mean ± SD of three independent experiments and at least fifty infected cells were analyzed for every condition during each independent experiment. Unpaired Student two-tailed *t* test was used to determine statistical significance. ns denotes not significant. Source data are provided as a Source Data file.

Supplementary Figure 2. Characterization of BioID-tagged EMC4 and EMC7. (related to Figure 4)

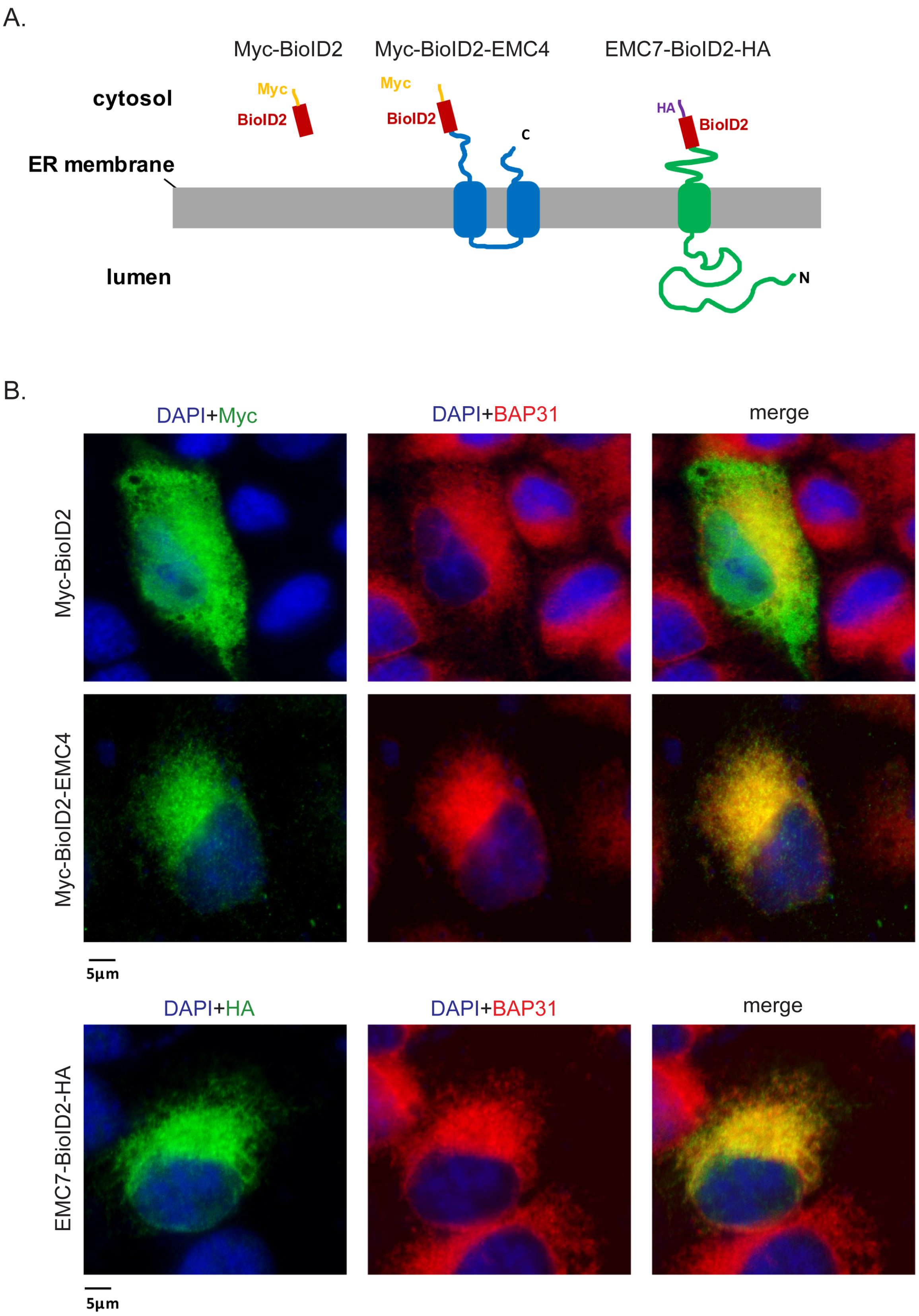

Supplementary Figure 2. Characterization of BioID2- tagged EMC4 and EMC7. (related to Figure 4)

A. Diagram of Myc-BioID2, Myc-BioID2-EMC4 and EMC7-BioID2-HA. B. CV-1 cells transfected with the indicated plasmid were fixed, stained with the indicated antibodies, and analyzed by epifluorescence widefield microscopy. This experiment was independently repeated for three times.

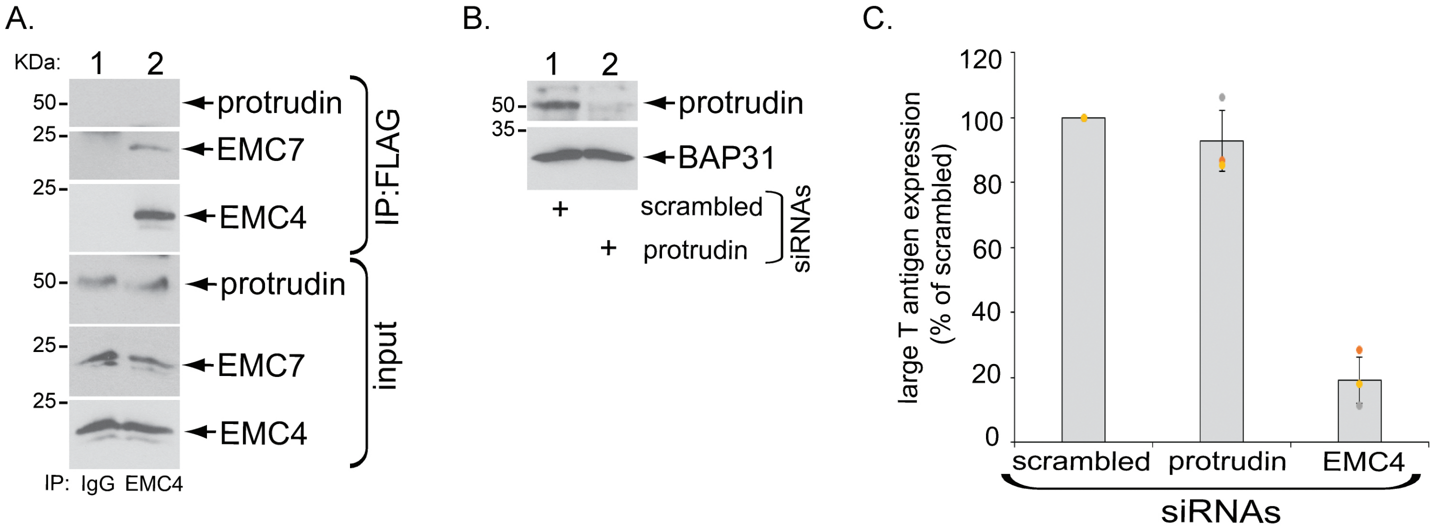

Supplementary Figure 3. Protrudin neither binds to EMC4 nor plays a role in SV40 infection. (related to Figure 4)

A. HEK 293T cells were lysed and the resulting extract subjected to immunoprecipitation using either rabbit IgG or EMC4 antibody. The precipitated materials were analyzed by SDS-PAGE followed by immunoblotting with indicated antibodies. This experiment was independently repeated for three times. B. CV-1 cells transfected with either scrambled or protrudin siRNA were lysed, the resulting extract subjected to SDS-PAGE, and immunoblotted with the indicated antibodies. This experiment was independently repeated for three times. C. CV-1 cells transfected with the indicated siRNAs were infected with SV40 (MOI ~ 0.5), fixed, and stained with large T antigen antibody. The percentages of T antigen positive cells were determined by using epifluorescence widefield microscopy. Values represent means ± SD from three independent experiments. Source data are provided as a Source Data file.

A.

scatter plot

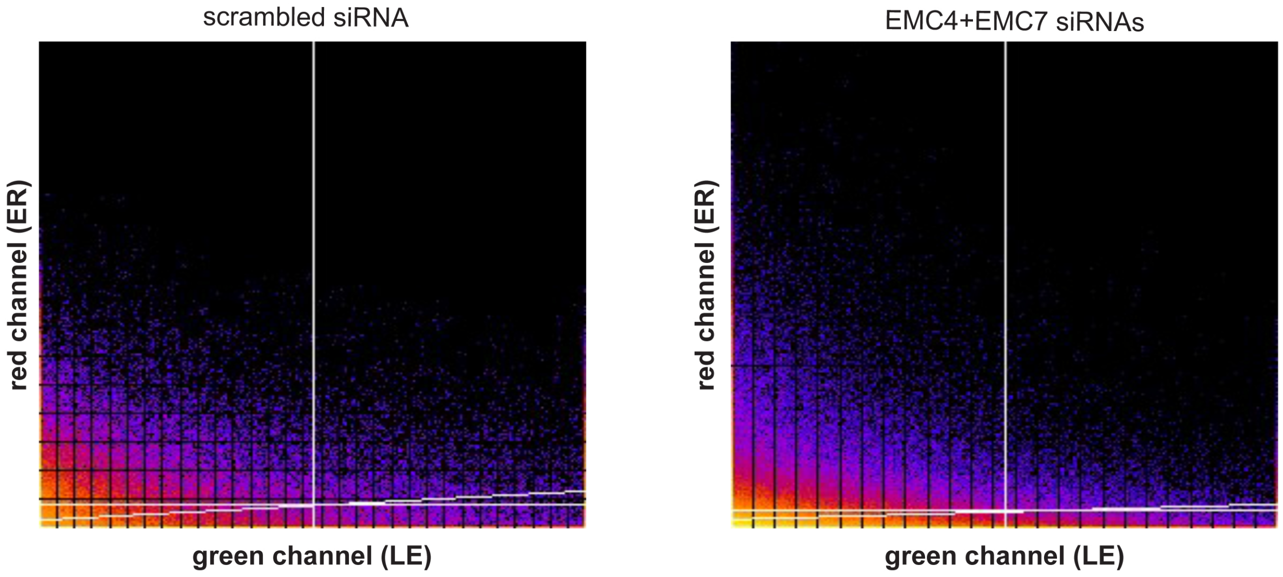

B.

co-localized pixel map (constant intensity)

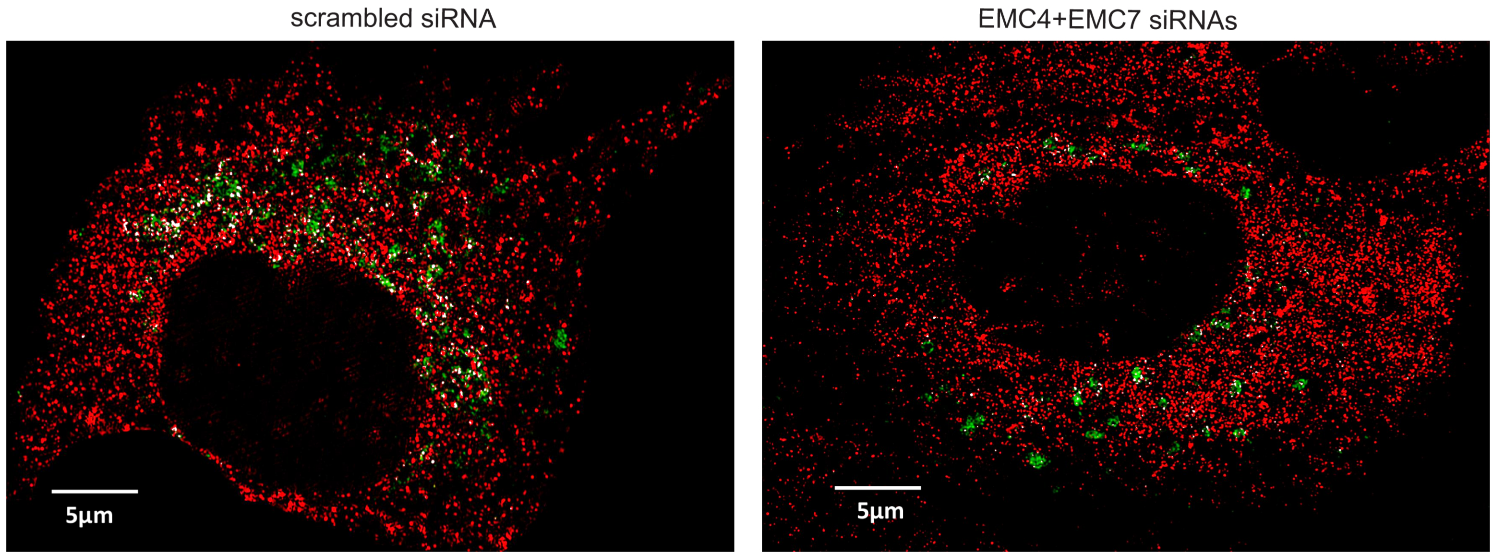

Supplementary Figure 4. Additional quantitation data supporting the role of EMC4 and EMC7 in mediating ER-LE co-localization. (Related to Figure 5)

A. Scatter plot showing pixel intensities corresponding to red and green channels in scrambled or EMC4+EMC7 siRNA-treated sample from Figure 5A. Scrambled siRNA treated sample shows more co-localized pixels above threshold (upper right quadrant) compared to the EMC4+EMC7 treated sample. B. Co-localized pixel map (in constant intensity) corresponding to the scrambled or EMC4+EMC7 siRNA-treated sample from Figure 5A showing more co-localized pixels (in white) in scrambled siRNA-treated sample when compared to the EMC4+EMC7 siRNA-treated sample. FIJI/ImageJ colocalization threshold plugin was used for the quantification analysis.

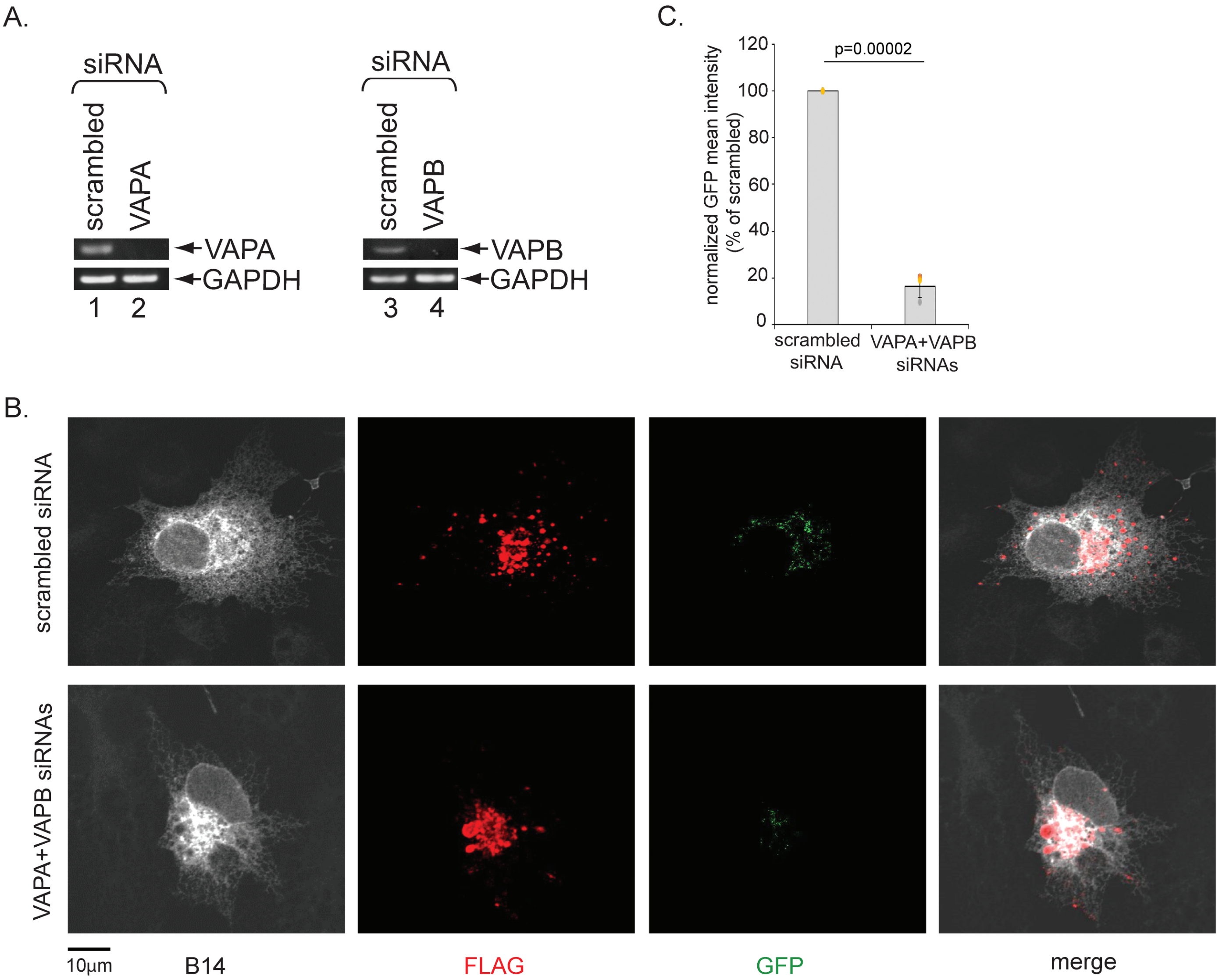

Supplementary Figure 5. Validation of the split GFP assay by VAPA and VAPB knock-down. (Related to Figure 6)
A. RT-PCR data showing the knockdown efficiency of the indicated siRNAs. B. GFP11-STARD3-FLAG and GFP (1-10)-B14 co-expressing CV-1 cells transfected with either scrambled or VAPA+VAPB siRNA were fixed, stained with B14 and FLAG antibody, and analyzed under confocal microscope for GFP signal. C. Mean GFP signal intensity from B was normalized with FLAG signal and quantified by FIJI/ImageJ. The image analysis was done on a single z-plane. Values represent means ± SD from three independent experiments. Unpaired Student two-tailed t test was used to determine statistical significance. Source data are provided as a Source Data file.

Supplementary Figure 6. EM analysis reveal EMC4 and EMC7 at the ER-LE contact. (related to Figure 6)

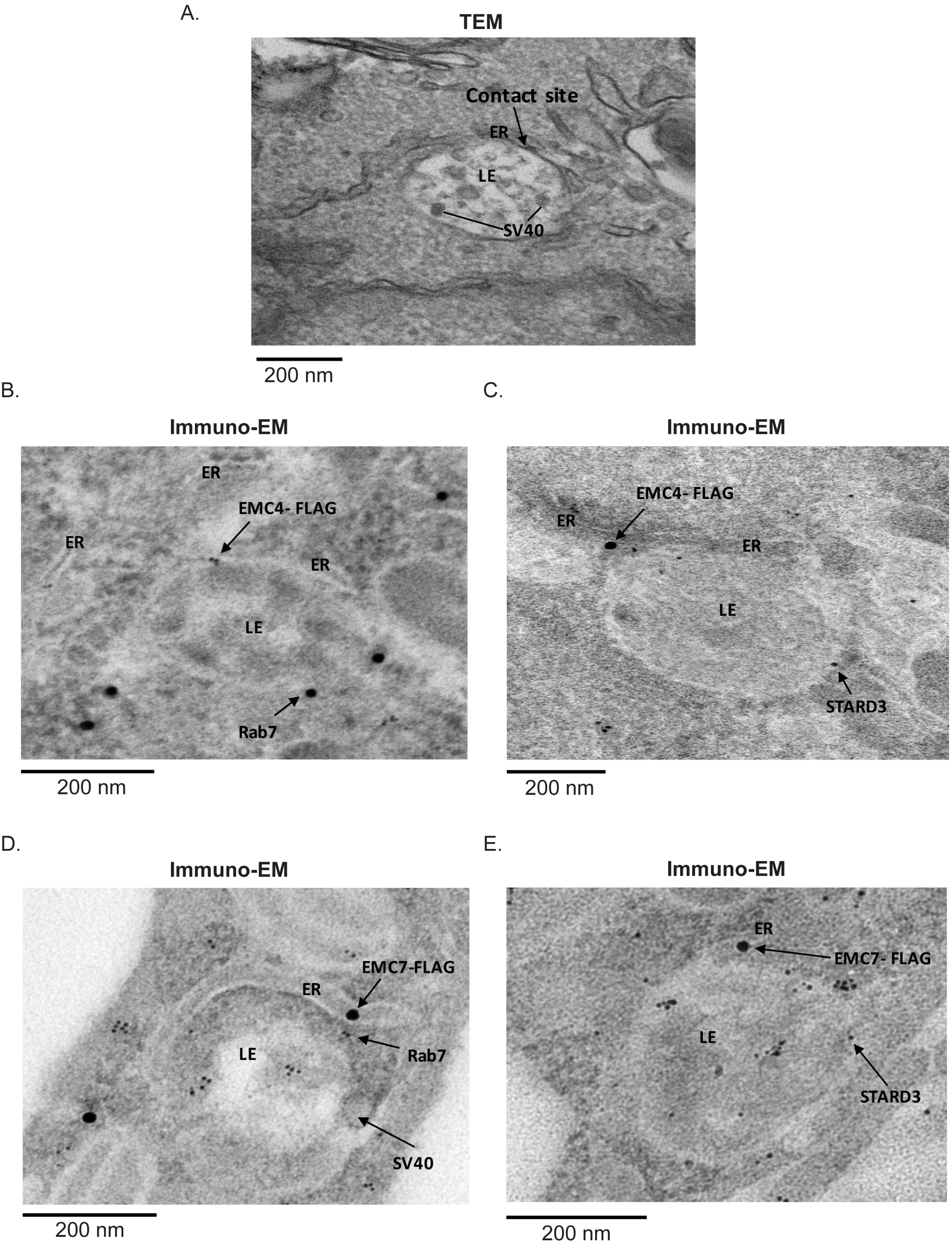

Supplementary Figure 6. EM analysis reveal EMC4 and EMC7 at the LE-ER contact. (Related to Figure 6)

A. Negative stained TEM image showing LE-ER contacts in SV40-infected CV-1 cell. B. Immunogold stained TEM image showing EMC4-FLAG at the site of LE-ER contact in COS-7 cells. Rab7 serves as the LE marker. 6 nm gold particle labels EMC4-FLAG and 15 nm gold particle labels Rab7. C. Immunogold stained TEM image showing EMC4-FLAG at the site of LE-ER contact in COS-7 cell. STARD3 serves as the LE marker. 15 nm gold particle labels EMC4-FLAG and 6 nm gold particle labels STARD3. D. Immunogold stained TEM image showing EMC7-FLAG at the site of LE-ER contact in COS-7 cell. Rab7 serves as the LE marker. 15 nm gold particle labels EMC7-FLAG and 6 nm gold particle labels Rab7. E. Immunogold stained TEM image showing EMC7-FLAG at the site of LE-ER contact in COS-7 cell. STARD3 serves as the LE marker. 15 nm gold particle labels EMC7-FLAG and 6 nm gold particle labels STARD3. All these experiments were independently repeated for three times.

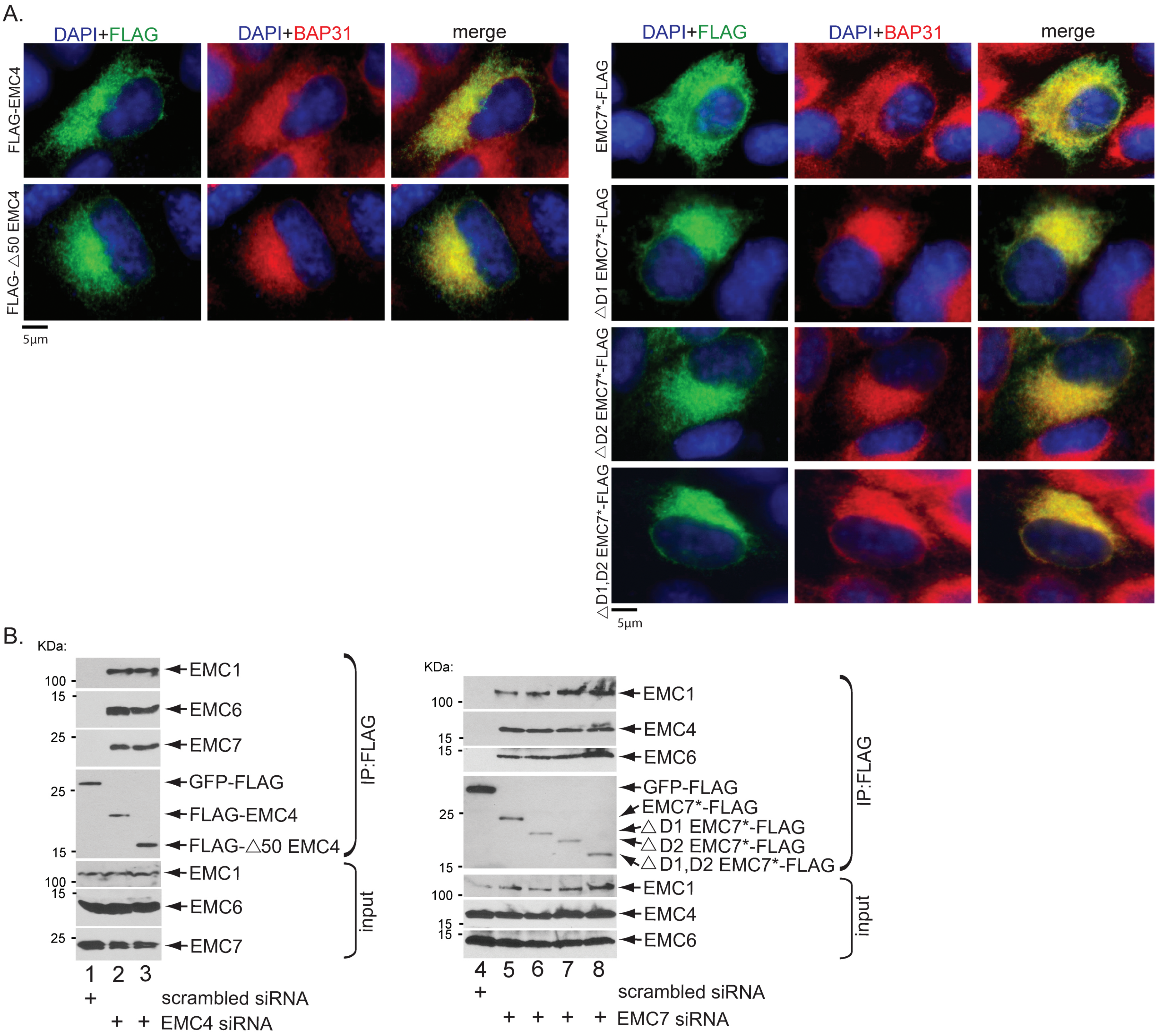

Supplementary Figure 7. Characterization of truncated EMC4 and EMC7 mutants. (Related to Figure 7)

A. CV-1 cells transfected with the indicated plasmid were fixed, stained with FLAG and BAP31 antibodies, and analyzed by epifluorescence widefield microscopy. This experiment was independently repeated for three times. B. HEK 293T cells transfected with the indicated siRNA and DNA construct were lysed, the resulting extract incubated with FLAG antibody-conjugated agarose beads, and the precipitated material subjected to SDS-PAGE and immunoblotting with the indicated antibodies. This experiment was independently repeated for three times. Source data are provided as a Source Data file.
