# Supplementary material for: Selective EMC subunits act as molecular tethers of intracellular organelles exploited during viral entry
Source: Nat Commun. 2020 Feb 28;11:1127. doi: 10.1038/s41467-020-14967-w (PMC7048770; doi:10.1038/s41467-020-14967-w)
Supplement: Supplementary file 3 — Reporting Summary [file 41467_2020_14967_MOESM3_ESM.pdf]

nature research

Corresponding author(s): Billy Tsai, Parikshit Bagchi

Last updated by author(s): Jan 22, 2020

Reporting Summary

Nature Research wishes to improve the reproducibility of the work that we publish. This form provides structure for consistency and transparency in reporting. For further information on Nature Research policies, see Authors & Referees and the Editorial Policy Checklist .

Statistics

For all statistical analyses, confirm that the following items are present in the figure legend, table legend, main text, or Methods section.

n/a	Confirmed	
☐	☒	The exact sample size (*n*) for each experimental group/condition, given as a discrete number and unit of measurement
☐	☒	A statement on whether measurements were taken from distinct samples or whether the same sample was measured repeatedly
☐	☒	The statistical test(s) used AND whether they are one- or two-sided<br>*Only common tests should be described solely by name; describe more complex techniques in the Methods section.*
☒	☐	A description of all covariates tested
☒	☐	A description of any assumptions or corrections, such as tests of normality and adjustment for multiple comparisons
☐	☒	A full description of the statistical parameters including central tendency (e.g. means) or other basic estimates (e.g. regression coefficient) AND variation (e.g. standard deviation) or associated estimates of uncertainty (e.g. confidence intervals)
☐	☒	For null hypothesis testing, the test statistic (e.g. *F*, *t*, *r*) with confidence intervals, effect sizes, degrees of freedom and *P* value noted<br>*Give P values as exact values whenever suitable.*
☒	☐	For Bayesian analysis, information on the choice of priors and Markov chain Monte Carlo settings
☒	☐	For hierarchical and complex designs, identification of the appropriate level for tests and full reporting of outcomes
☐	☒	Estimates of effect sizes (e.g. Cohen's *d*, Pearson's *r*), indicating how they were calculated

Our web collection on statistics for biologists contains articles on many of the points above.

Software and code

Policy information about availability of computer code

Data collection	Confocal and SIM images were taken with NIS-Elements AR sofware (version 5.02.01) from Nikon.
Data analysis	FIJI from NIH (version 1.52p)

For manuscripts utilizing custom algorithms or software that are central to the research but not yet described in published literature, software must be made available to editors/reviewers. We strongly encourage code deposition in a community repository (e.g. GitHub). See the Nature Research guidelines for submitting code & software for further information.

Data

Policy information about availability of data

All manuscripts must include a data availability statement. This statement should provide the following information, where applicable:

- Accession codes, unique identifiers, or web links for publicly available datasets
- A list of figures that have associated raw data
- A description of any restrictions on data availability

The data that support the findings of this study are available from the corresponding author upon reasonable request.

Field-specific reporting

Please select the one below that is the best fit for your research. If you are not sure, read the appropriate sections before making your selection.

☒ Life sciences ☐ Behavioural & social sciences ☐ Ecological, evolutionary & environmental sciences

For a reference copy of the document with all sections, see nature.com/documents/nr-reporting-summary-flat.pdf

Life sciences study design

All studies must disclose on these points even when the disclosure is negative.

Sample size	Sample size is generally important in clinical and epidemiological studies. As this study does not belong in this category, the sample size is not applicable for this manuscript.
Data exclusions	No data exclusion
Replication	All experiments were replicated at least three times and all attempts of replication were successful.
Randomization	GFP-expressing cells and scrambled siRNA-transfected cells were grouped as control. Cells expressing protein of interest (such as EMC4 or EMC7) and cells transfected with a siRNA against a protein of interest (such as EMC4 or EMC7) were grouped as test samples.
Blinding	Under the microscope, GFP-expressing cells (control) is visually distinct from cells expressing the protein of interest (test) which are ER membrane proteins; for this reason, it is not possible to blind this experiment. In the knockdown studies, instead of blinding, data were confirmed independently by other investigators in the lab.

Reporting for specific materials, systems and methods

We require information from authors about some types of materials, experimental systems and methods used in many studies. Here, indicate whether each material, system or method listed is relevant to your study. If you are not sure if a list item applies to your research, read the appropriate section before selecting a response.

Materials & experimental systems

n/a	Involved in the study	
☐	☒	Antibodies
☐	☒	Eukaryotic cell lines
☒	☐	Palaeontology
☒	☐	Animals and other organisms
☒	☐	Human research participants
☒	☐	Clinical data

Methods

n/a	Involved in the study	
☒	☐	ChIP-seq
☒	☐	Flow cytometry
☒	☐	MRI-based neuroimaging

Antibodies

Antibodies used

1) Anti-BAP31; Thermo Fisher Scientific; Cat# MA1-34492; RRID: AB_2537133; Working dilution 1:3000 for WB and 1:250 for IF
2) Anti-EMC1; Abgent; Cat# AP10226b; RRID: AB_10817224; Working dilution 1:2000 for WB
3) Anti-EMC4 (TMEM85); Abcam; Cat# ab184162; RRID: N/A; Working dilution 1:4000 for WB
4) Anti-EMC6 (TMEM93); Aviva Systems Biology; Cat# ARP44679_P050; RRID: AB_2048477; Working dilution 1:2000 for WB
5) Anti-EMC7; Thermo Fisher Scientific; Cat# PA5-52688; RRID: AB_2641011; Working dilution 1:300 for WB
6) Anti-FLAG clone M2; Sigma Aldrich; Cat# F1804; RRID: AB_262044; Working dilution 1:5000 for WB, 1:500 for IF and 1:20 for Immuno-EM
7) Anti-FLAG; Sigma Aldrich; Cat# F7425; RRID: AB_439687; Working dilution 1:5000 for WB, 1:500 for IF and 1:20 for Immuno-EM
8) Anti-GFP; Proteintech; Cat# 660002-1-Ig; RRID: N/A; Working dilution 1:10000 for WB
9) Anti-HA; Proteintech; Cat# 51064-2-AP; RRID: AB_11042321; Working dilution 1:5000 for WB and 1:500 for IF
10) Anti-SV40 large T antigen; Santa Cruz Biotechnology; Cat# sc-147; RRID: AB_628305; Working dilution 1:100 for IF
11) Anti-Hsp90; Santa Cruz Biotechnology; Cat# sc-13119; RRID: AB_675659; Working dilution 1:2000 for WB
12) Anti-Stx18; Santa Cruz Biotechnology; Cat# sc-293067; RRID: AB_10647235; Working dilution 1:1000 for WB
13) Anti-SV40 VP1 antibody (mouse monoclonal); Gift from Walter Scott (University of Miami) and the antibody was generated by them; Cat# N/A; Working dilution 1:2000 for WB and 1:500 for IF
14) Anti-SV40 VP1 antibody (rabbit polyclonal); Gift from Harumi Kasamatsu (UCLA) and the antibody was generated by them ; Cat# N/A; Working dilution 1:2000 for WB and 1:500 for IF
15) Anti-BiP; Abcam; Cat# ab32618; RRID: AB_732737; Working dilution 1:1000 for WB
16) Anti-SV40 VP2/3; Abcam; Cat# ab53983; RRID: AB_946339; Working dilution 1:500 for IF
17) Anti-CTA; EMD Millipore; Cat# 227040; RRID: AB_211712; Working dilution 1:2000 for WB
18) Anti-Rab7 (mouse monoclonal); Sigma Aldrich; Cat# R8779; RRID: AB_609910; Working dilution 1:500 for WB, 1:100 for IF and 1:20 for Immuno-EM
19) Anti-Rab7 (rabbit monoclonal); Cell Signaling Technology; Cat# 9367; RRID: AB_1904103; Working dilution 1:1000 for WB and 1:200 for IF
20) Anti-Protrudin (ZFYVE27); Proteintech; Cat# 12680-1-AP; RRID: AB_10640298; Working dilution 1:1000 for WB
21) Anti-EEA1 (rabbit monoclonal); Cell Signaling Technology; Cat# 3288; RRID: AB_2096811; Working dilution 1:500 for IF
22) Anti-Myc; Gift from Kristen Verhey (University of Michigan); Santa Cruz Biotechnology; Cat# sc-40; RRID: AB_627268;

Working dilution 1:1000 for WB and 1:200 for IF
23) Anti-Rab5; Abcam; Cat# ab18211; RRID: AB_470264; Working dilution 1:200 for WB
24) Anti-STARD3 (MLN64); Abcam; Cat# ab3478; RRID: AB_303838; Working dilution 1:20 for Immuno-EM
25) Anti-DnaJB14; Proteintech; Cat# 16501-1-AP; RRID: AB_2094414; Working dilution 1:10000 for WB
26) F(ab') 2 Fragment of Goat-anti-Mouse IgG (H&L) (EM grade 15nm); Electron Microscopy Sciences; Cat # 25377; RRID: N/A; Working dilution 1:25 for Immuno-EM
27) F(ab') 2 Fragment of Goat-anti-Rabbit IgG (H&L) (EM grade 6nm); Electron Microscopy Sciences; Cat # 25364; RRID: N/A; Working dilution 1:25 for Immuno-EM
28) Goat-anti-Mouse IgG (H&L) (EM grade 6nm); Electron Microscopy Sciences; Cat # 25124; RRID: N/A; Working dilution 1:25 for Immuno-EM
29) Goat-anti-Rabbit IgG (H&L) (EM grade 15nm); Electron Microscopy Sciences; Cat # 25113; RRID: N/A; Working dilution 1:25 for Immuno-EM

Validation

1) Anti-BAP31; Validation statement on the manufacturer's website stating application in Western Blot, Immunofluorescence and reactive against human and non-human primates.
2) Anti-EMC1; Validation statement on the manufacturer's website stating application in Western Blot and reactive against human. Validated previously for Western Blot and reactive against monkey species (Bagchi et. al., Elife, 2016).
3) Anti-EMC4 (TMEM85); Validation statement on the manufacturer's website stating application in Western Blot and reactive against human. Validated for Western Blot and reactive against monkey species in present manuscript.
4) Anti-EMC6 (TMEM93); Validation statement on the manufacturer's website stating application in Western Blot and reactive against human. Validated for Western Blot and reactive against monkey species in present manuscript.
5) Anti-EMC7; Validation statement on the manufacturer's website stating application in Western Blot and reactive against human. Validated for Western Blot and reactive against monkey species in present manuscript.
6) Anti-FLAG clone M2; Validation statement on the manufacturer's website stating application in Western Blot, Immunofluorescence, Immunoprecipitation.
7) Anti-FLAG; Validation statement on the manufacturer's website stating application in Western Blot, Immunofluorescence, Immunoprecipitation.
8) Anti-GFP; Validation statement on the manufacturer's website stating application in Western Blot, Immunofluorescence.
9) Anti-HA; Validation statement on the manufacturer's website stating application in Western Blot, Immunofluorescence.
10) Anti-SV40 large T antigen; Validation statement on the manufacturer's website stating application in Western Blot, Immunofluorescence.
11) Anti-Hsp90; Validation statement on the manufacturer's website stating application in Western Blot and reactive against human. Validated previously for Western Blot and reactive against monkey species (Bagchi et. al., Elife, 2016).
12) Anti-Stx18; Validation statement on the manufacturer's website stating application in Western Blot, Immunoprecipitation and reactive against human. Validated for Western Blot and reactive against monkey species in present manuscript.
13) Anti-SV40 VP1 antibody (mouse monoclonal); Validated previously for Western Blot, Immunofluoresence (Inoue and Tsai, PLOS Pathogens, 2011; Bagchi et. al., Journal of Virology, 2015; Bagchi et. al., Elife, 2016).
14) Anti-SV40 VP1 antibody (rabbit polyclonal); Validated previously for Western Blot, Immunofluoresence (Inoue and Tsai, PLOS Pathogens, 2011; Bagchi et. al., Journal of Virology, 2015; Bagchi et. al., Elife, 2016).
15) Anti-BiP; Validation statement on the manufacturer's website stating application in Western Blot and reactive against human. Validated previously for Western Blot and reactive against monkey species (Bagchi et. al., Elife, 2016).
16) Anti-SV40 VP2/3; Validation statement on the manufacturer's website stating application in Western Blot, Immunofluorescence.
17) Anti-CTA; Validation statement on the manufacturer's website stating application in Western Blot.
18) Anti-Rab7 (mouse monoclonal); Validation statement on the manufacturer's website stating application in Western Blot, Immunofluorescence and reactive against human and monkey.
19) Anti-Rab7 (rabbit monoclonal); Validation statement on the manufacturer's website stating application in Western Blot, Immunofluorescence and reactive against human and monkey.
20) Anti-Protrudin (ZFYVE27); Validation statement on the manufacturer's website stating application in Western Blot, Immunofluorescence and reactive against human. Validated for Western Blot and reactive against monkey species in present manuscript.
21) Anti-EEA1 (rabbit monoclonal); Validation statement on the manufacturer's website stating application in Immunofluorescence and reactive against human. Validated for immunofluoresence and reactive against monkey species in present manuscript.
22) Anti-Myc; Validated previously for Western Blot and Immunofluoresence (Verhey et. al., Journal of Cell Biology, 1998).
23) Anti-Rab5; Validation statement on the manufacturer's website stating application in Western Blot and reactive against human.
24) Anti- STARD3 (MLN64); Validation statement on the manufacturer's website stating application in Western Blot, ICC/IF, IHC-P, ICC, IP, Inhibition Assay and reactive against human, Mouse, Rat, Hamster, Cow. Validated for immuno-EM and reactive against monkey species in present manuscript.
25) Anti-DnaJB14; Validation statement on the manufacturer's website stating application in Western Blot, IP, IF and reactive against human, mouse, rat. Validated previously for IF and reactive against monkey species (Bagchi et. al., J. Virol., 2015).

Eukaryotic cell lines

Policy information about cell lines

Cell line source(s)	CV-1, HEK293T, COS-7 from American Type Culture Collection (ATCC)
Authentication	Cells from ATCC are authenticated by morphology, karyotyping and PCR based approaches according to ATCC.
Mycoplasma contamination	All cells are negative from Mycoplasma contamination
Commonly misidentified lines (See ICLAC register)	No commonly misidentified cell lines were used
